# The efficacy of 3D personalized insoles in moderate adolescent idiopathic scoliosis: a randomized controlled trial

**DOI:** 10.1186/s12891-022-05952-z

**Published:** 2022-11-14

**Authors:** Bin Wang, Yue Sun, Xiaoqi Guo, Jiangang Cao, Haoyuan Lu, Wei Chen, Jie Chen, Qian Zhu, Chong Zhang, Ming Zhang, Feilong Zhu

**Affiliations:** 1 The Affiliated Xuzhou Rehabilitation Hospital of Xuzhou Medical University, Xuzhou Rehabilitation Hospital, Xuzhou, China; 2 School of Sports Medicine and Rehabilitation, Beijing Sport University, Beijing, China; 3 Department of Rehabilitation Medicine, Xuzhou Central Hospital, The Xuzhou Clinical College of Xuzhou Medical University, Xuzhou, China; 4 College of Physical Education and Sports, Beijing Normal University, Beijing, China

**Keywords:** Adolescent idiopathic scoliosis, Insole, Bracing, Exercises, Rehabilitation

## Abstract

**Background:**

Bracing and exercise methods were used in scoliosis rehabilitation and proven effective. There was little evidence about the efficacy of insoles on scoliosis.

**Objective:**

This study aimed to investigate the effects of 3D personalized insoles on curve magnitude, postural stability, and quality of life (QOL) in moderate adolescent idiopathic scoliosis (AIS) patients.

**Methods:**

Thirty-six volunteers with adolescent idiopathic scoliosis, who had moderate curves (20°-45°), were randomly divided into the experimental and control groups. The control group received traditional rehabilitation with bracing and exercises, and the experimental group received the insole interventions in addition to traditional rehabilitation. The outcome measures were Cobb angle, angle of trunk rotation (ATR), postural stability, and quality of life (Scoliosis Research Society-22 questionnaire). Measurements were conducted at baseline examination, two months and six months.

**Results:**

After two and six months of treatment, the Cobb angle and ATR in both groups were significantly decreased as compared with the baseline (*p* < 0.05), but no significant group difference in Cobb angle and ATR was found in the study (*p* > 0.05). There was a significant difference in the sagittal balance index at six months compared to the control group (*p* < 0.05), and a significant difference in the coronal balance index was observed at six months compared to baseline in the experimental group (*p* < 0.05). Quality of life did not change in either group (*p* > 0.05).

**Conclusion:**

Combining bracing with exercise in patients with moderate AIS is effective. 3D personalized insoles cannot reduce the Cobb angle and angle of trunk rotation of patients with moderate AIS but might have the potential to improve postural stability.

## Introduction

Adolescent idiopathic scoliosis (AIS) is a spinal curvature with a Cobb angle greater than 10 degrees with an average onset age of 10 to 18 years old, affecting up to 4% of adolescents worldwide [1–4]. Individuals with scoliosis are often accompanied by trunk deformity, spinal rigidity, postural changes, unsteadiness, and gait variations, which can cause pain and reduce quality of life if untreated, especially in patients with greater curvature [5–7].

Current treatment modalities for AIS include physical therapy, exercises, bracing, and surgery according to the patient’s Cobb angle [1, 4, 8, 9]. For scoliosis with a Cobb angle of less than 10°, close follow-up and postural education should be provided. In patients where the curve progresses to 10°-20°, scoliosis-specific exercises have been recommended as the first line of treatment for small curves [4, 10]. Surgery is recommended for adolescents with a curve that have a Cobb angle greater than 45° or a slightly smaller curvature aggravated by more than 6° per year after brace interventions^1^. For moderate curves (Cobb angle 20°-45°), the conservative treatment is combined spinal braces and exercise approaches. The effectiveness of bracing combined with exercise in the management of AIS was reported by a prospective randomized controlled study, which demonstrated that the spinal orthosis combined with exercise significantly decreased the Cobb angle [11]. Bracing in AIS can prevent curve progression, which is based on the principle of external forces guiding the growth of the spine and show the effectiveness of bracing in juveniles [1, 12]. The mechanism of the spinal brace reducing the curve magnitude is to change the patient’s posture by keeping the trunk in a fixed (corrected) position. In contrast, exercises aim to create behavioral and automatic changes of movement and posture through the use of different motor control strategies [13, 14].

Although combined bracing and exercises is effective in reducing progression and preventing surgery in AIS patients, what cannot be ignored is that AIS patients are often accompanied by obliquity of the pelvis and imbalance in lower extremity biomechanics, such as flat foot and leg inequality [15, 16]. Currently, orthopedic spinal braces are mainly divided into cervico-thoraco-lumbo-sacral-orthosis (CTLSO) and thoraco-lumbo-sacral-orthosis (TLSO) [17], and the effectiveness has been objectively proven through imaging assessment and finite element analysis (FEA) for the biomechanics evaluation [12, 18]. But such orthoses mainly lie in changing spinal curvature rather than foot abnormality correction. Insoles play a positive role in improving biomechanics and compensation, regulating abnormal posture, dispersing plantar pressure and adjusting gait, etc. [19]. Additionally, insoles have the advantages of simplicity, convenient wearing, non-invasiveness, and good patient compliance [20]. Some studies reported that insoles were helpful to foot disorders [21], improving neurological disabilities and inducing spinal postural changes [22]. Noll assumed that proprioceptive insoles could affect posture and gait function by regulating the sensory structure of the sole of the feet, but no evidence of any statistically significant effect of proprioceptive insoles on spinal curvature in patients with slight idiopathic scoliosis was observed [23]. The probable reason may be that proprioceptive insoles were used in this study, and absence of sufficient mechanical compensations and corrections [23]. Besides, slight scoliosis may not have significant abnormal biomechanical manifestations. We still have reasons to believe that correcting abnormal biomechanics of the feet, such as supporting the arch, controlling inversion and eversion of the heel, and distributing local plantar pressure, may contribute to improving AIS. Although the effects of foot orthotics on biomechanics of the knee, ankle and toe were well described, we still know little about the effects on spinal curvature [24–26], and it is worthy of continuing to be explored.

Hence, this randomized controlled study was conducted to determine the effectiveness of insoles in the treatment of moderate AIS, and we hypothesized that adding 3D personalized insoles intervention to the traditional treatment (bracing and exercise) was better than the traditional treatment alone in patients with moderate AIS.

## Methods

This study was a single-center, single-blinded randomized controlled trial and was performed in line with the principles of the Declaration of Helsinki. This study protocol was registered in the World Health Organization International Clinical Trials Registry Platform of the China Clinical Trial Register under registration No. ChiCTR2000035205 (03/08/2020). Results of the randomized controlled trial were reported in accordance with CONSORT 2010 guidelines [27]. The ethics committee of the Affiliated Xuzhou Rehabilitation Hospital of Xuzhou Medical University reviewed and approved the study protocol (Ethics number: XKYL2019014). All participants and their legal guardians provided written informed assent and consent prior to participation.

### Participants

#### Inclusion and exclusion criteria

Patients diagnosed with AIS were enrolled at the Affiliated Xuzhou Rehabilitation Hospital of Xuzhou Medical University (Jiangsu, China) between August 2020 and October 2021. An experienced orthopedist identified them. The inclusion criteria were: (1) age range 10 to 18 years regardless of gender; (2) presence of moderate scoliosis (20°≤Cobb angle ≤ 45°) detected by X-rays; (3) with relevant growth potential and Risser sign ≤ grade 3; (4) right limb dominance; (5) no prior scoliosis-related treatments; (6) be willing to take X-rays following the orthopedist’s recommendations; (7) no other congenital or known musculoskeletal, neuromuscular, nervous system, endocrine, psychiatric, infectious, traumatic disease; (8) no disorder or surgery history of the spine, lower limbs, and the feet; (9) be able to walk normally without assistance. The exclusion criteria were as follows: (1) non-idiopathic scoliosis; (2) with different abnormalities affecting normal locomotion; (3) previously treated patients for scoliosis; (4) failure to follow study instructions; (5) having contraindications to exercises.

#### Sample size estimation

We used the primary outcome of the Cobb angle to determine the sample size according to the previous research [14, 23]. The sample size was determined using the software PASS (version 15.0.1) with the input: repeated measure analysis of variance (ANOVA) within and between interactions, the statistical power of 0.8, a medium effect size of 0.3, a significance level of 0.05 and dropout rate of 10%. Consequently, the estimated sample size was 14 subjects per group.

#### Blinding and allocation

We assessed 78 volunteers for eligibility. After screening for inclusion and exclusion criteria, 36 volunteers were entered into the trial. After a simple randomization procedure (computerized random numbers), thirty-six participants were randomly assigned to the experimental or control group (ratio 1:1) following a simple randomization procedure (computerized random numbers). The randomization sequence was created by an investigator with no clinical involvement in this study using a computerized program (IBM SPSS version 25 software). The allocation sequence was concealed using sequentially numbered sealed opaque envelopes. In addition, the outcome assessor was blinded to the group assignment. Owing to the nature of the interventions, it was not possible to blind patients to the treatment allocation.

### Interventions

We were trying to avoid the progression of scoliosis due to inappropriate interventions and treatments in these participants associated with this trial. We followed the general treatment guidelines of AIS [28] and suggestions from the Ethics Committee, and participants in the experimental group and control group all received the traditional treatments with brace and exercises. The insole intervention was specifically added to the experiment group. These interventions were individually developed for participants to ensure the best possible clinical effectiveness.

#### Interventions-control group

**Fig. 1:**
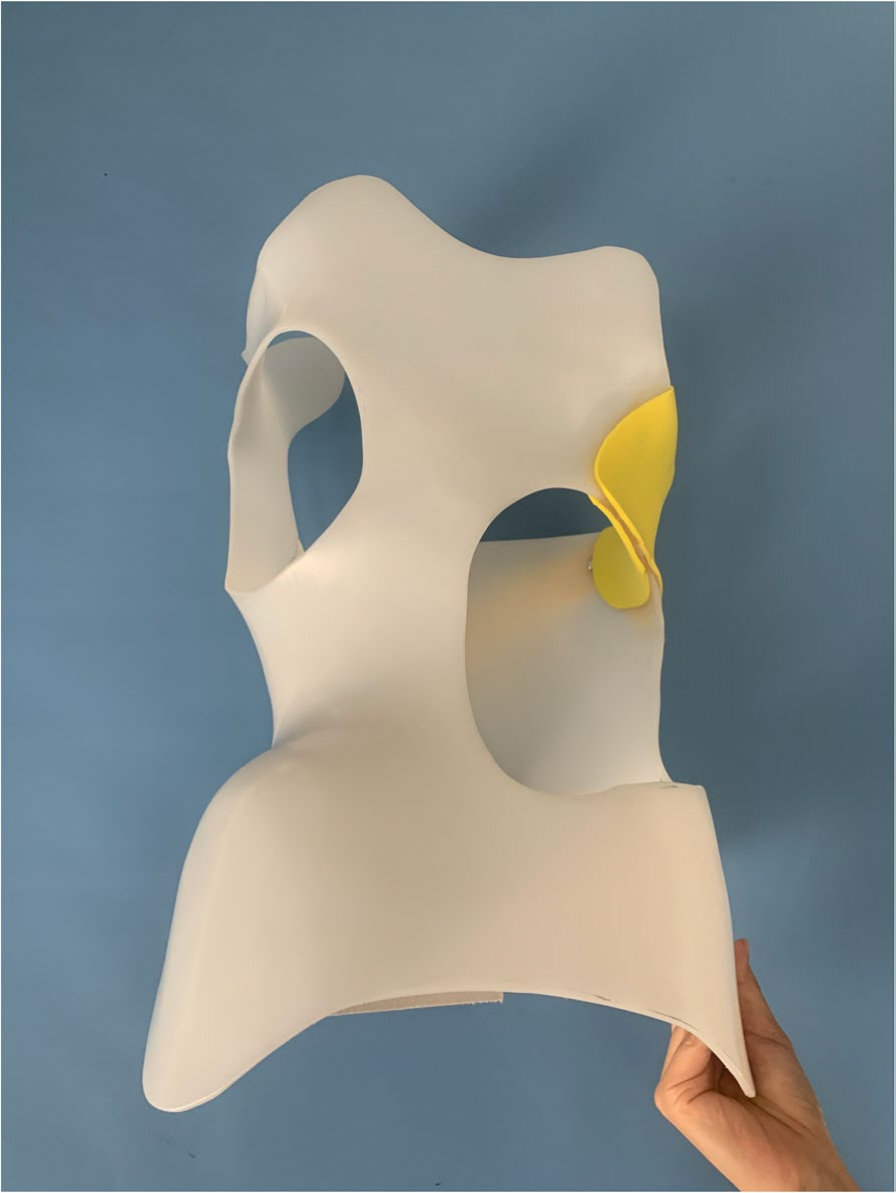
The thoraco-lumbo-sacral spinal brace

The control group received the standard of care, including bracing and exercises. Spinal bracing is one of the most essential traditional treatment options in patients with moderate AIS (Cobb angle 20°-45°) [29]. A professional orthopedic technician made the brace by applying the augmentation-release system of three-point corrective force and two-point anti-rotational force coupling. The thoraco-lumbo-sacral spinal brace was used in this study **(**Fig. 1**)**, which was designed based on the individually-oriented, rigid, active, custom-made, and three-dimensional concept [30–32]. The participants were instructed to wear the brace for 22–23 h daily, and removal of the brace was allowed during bathing and exercises.

The specific exercise program was as follows. Physiotherapists specializing in scoliosis performed traditional stretching and stabilization exercises first and adjusted them accordingly at follow-up. The guardians of these participants were taught how to perform these exercises at home properly. Traditional exercise programs for scoliosis included breathing exercises, posture training, spinal flexibility exercises, stretching exercises for the concave side of the curve, and strengthening exercises for the convex side [30, 33]. These specific exercises for scoliosis were performed at least three times a week for half an hour each time. The scoliosis-specific exercises required close supervision and repeated clinical visits from professionals, and lengthy sessions. Therefore, we required guardians to upload photos or videos during exercises regularly to improve patient compliance with the exercise and the efficacy of treatment.

#### Interventions-experimental group

**Fig. 2:**
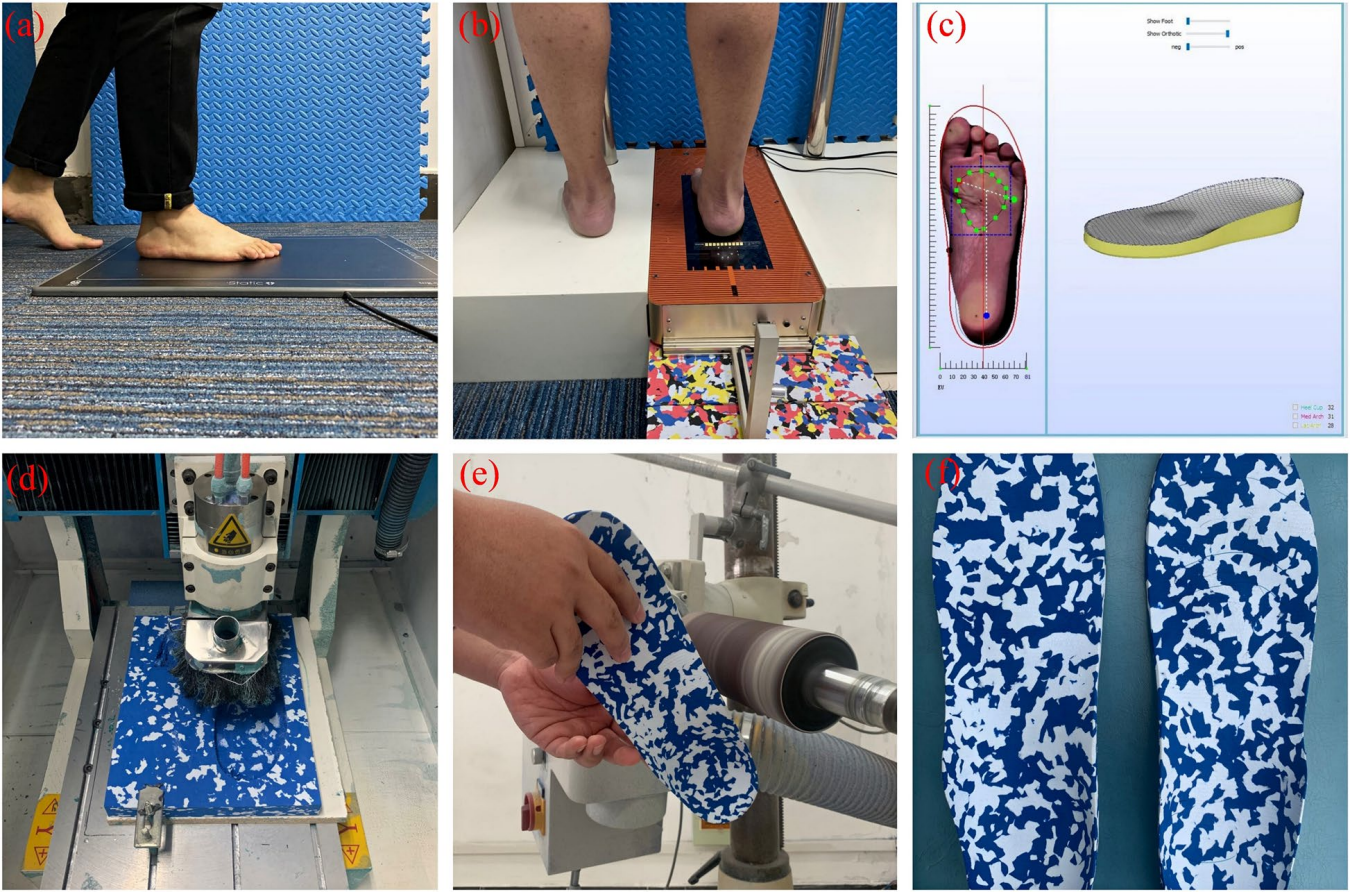
The process of 3D personalized insoles production. **a** Plantar pressure distribution measurement; **b** 3D foot scanner measurement; **c** Computer-aided design (CAD); **d** Insoles engraving; **e** The rough insoles was polished; **f** 3D personalized insoles

In the experimental group, participants received insoles intervention in addition to standard treatments with brace and exercises as in the control group. The participants were invited to the Foot and Ankle Biomechanics Laboratory of the Affiliated Xuzhou Rehabilitation Hospital of Xuzhou Medical University (Jiangsu, China). The insole used in this study was a computer-aided design of 3D engraved insole, which was a completely personalized and customized insole. Insoles were made of Ethylene Vinyl Acetate Copolymer (EVA) material. Procedures were briefly described as follows: (1) Assessment of lower extremity biomechanics: A professional orthopedic technician assessed the participant’s neutral and resting heel position in the static standing position, tibial torsion angle, bilateral hip internal and external rotation mobility, and the difference in lower limbs length according to the Najjarine assessment system developed by Professor Abbie Najjarine. (2) Static and dynamic plantar pressure distribution measurement (Fig. 2a): The GaitScan pedal pressure system (The Orthotic Group, Markham, Ontario, Canada) was used to obtain some data such as the gait characteristic, plantar pressure distribution, balance, and direction of movement during standing and walking. (3) 3D foot scanner measurement **(**Fig. 2b**)**: The LSR Laser Foot Scanner system (Vismach Technology Ltd) was used to extract foot feature parameters based on plantar, dorsal, medial, lateral, toe, and heel views of the foot. The foot parameters used in the study were foot length, foot width, heel width, arch length, 1–5 metatarsal width, and arch index (AI). (4) Computer-aided design (CAD) **(**Fig. 2c**)**: The design of 3D insoles was based on the results of the biomechanical assessment of the lower limbs and machine scanning. We increased the stress points or cut off the load-free parts of the insole, including personalized increased arch support, adding wedge blocks with different heights to the heel for adjusting the heel in a neutral position, controlling the degree of forefoot inversion and eversion, and bilateral lower limb length compensation, etc. (5) Engraving (Fig. 2d): We imported the designed insole program into the computer engraving program for conversion and set the engraving method and precision, and connected the engraving machine for model engraving. (6) Polishing **(**Fig. 2e**)**: The rough insoles needed to be polished after engraving. (7) Trying on and delivery: Participants put on the insoles and were observed whether the biomechanical abnormalities of the lower limbs in the standing position and during walking were improved. Insoles were used daily for a minimum of eight hours per day. The insoles are shown in Fig. 2f.

### Outcomes and follow-up

Demographic information of the participants, including age, gender, height, body mass, Risser sign, and curve pattern, were collected by the same assessor. The skeletal immaturity based on the Risser stages and curvature pattern classified by the King classification system were recorded for each participant at the first visit. The study’s outcome measures, including the Cobb angle, angle of trunk rotation (ATR), coronal balance index, sagittal balance index, and SRS-22 questionnaire, were followed up at two and six months of intervention. The two-month follow-up period was chosen because these patients needed to be re-evaluated for appropriate brace and exercise adjustments at two months. The six-month follow-up was selected as the insole may have a distortion during long-term use due to weight-bearing, which may affect the treatment effect.

#### Primary outcomes

##### Cobb angle

Since the first description by John Cobb in 1948, the Cobb angle measurement has been the gold standard method to diagnose and evaluate spinal curvature magnitude [34]. In this study, we still used the Cobb angle method as the primary outcome for monitoring scoliosis progression and assessing the curve magnitude. The Cobb angle was measured from anterior-posterior longitudinal X-ray films, and the curve with the largest Cobb angle was selected as the primary curve in the patients.

#### Secondary outcomes

##### Angle of trunk rotation (ATR)

The angle of trunk rotation was measured by using a Scoliometer in Adam’s forward bend test, which has been shown to have good consistency, inter-rater, and test-retest reliability [35].

##### **Coronal balance index and sagittal balance index**

**Fig. 3:**
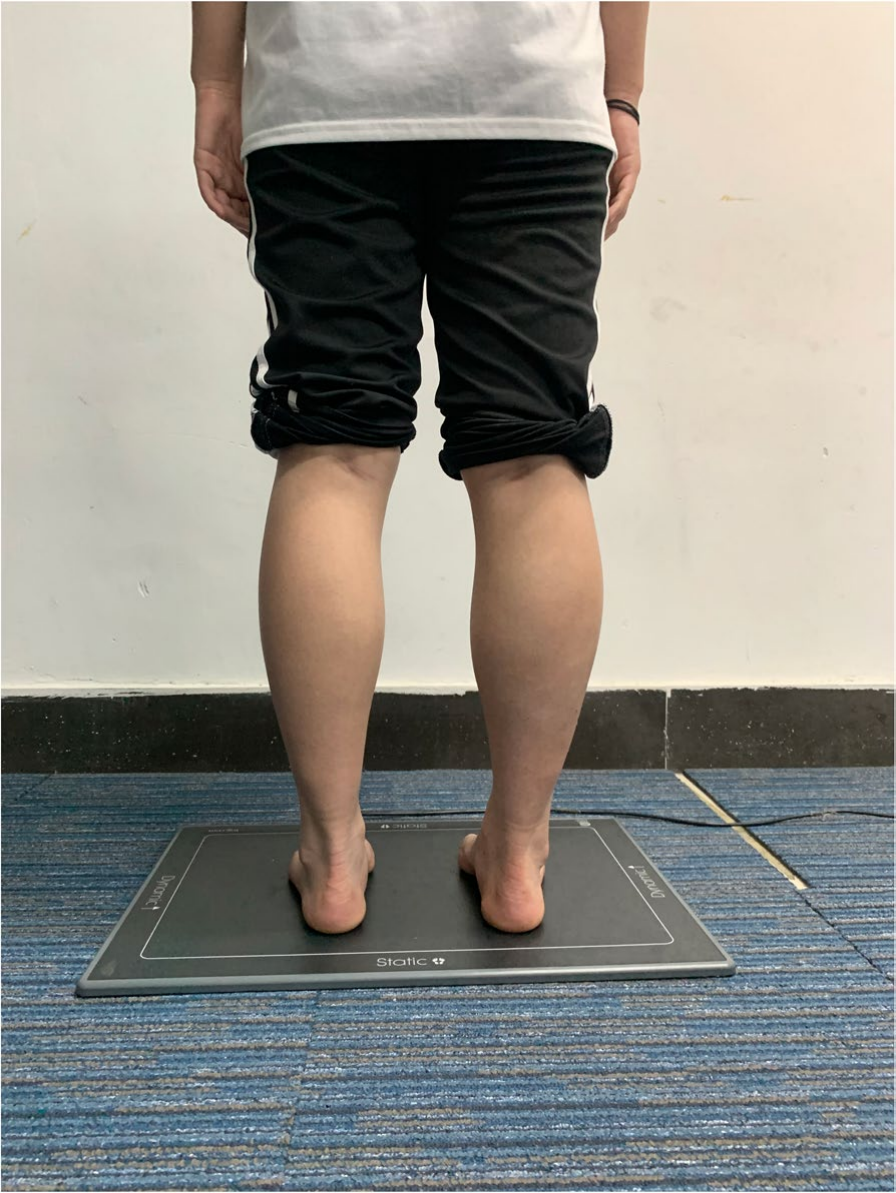
Balance and postural stability were assessed using a plantar pressure distribution method

In this study, balance and postural stability in patients with AIS were assessed using a plantar pressure distribution method (Fig. 3). The plantar pressure distribution was measured by the GaitScan (The Orthotic Group, Markham, Ontario, Canada) pedal pressure system during level barefoot standing. The GaitScan pedal pressure system consists of a 578*418*12 mm floor mat incorporating 4096 sensors on a single mat, and the system samples at a rate of 125 Hz. During testing, all measurements were performed barefoot in a relaxed standing position with the eyes looking straight forward. The participants were instructed to stand on the pressure plate and remain stable. The percentage of impulse under the left and right foot, forefoot, and hindfoot was collected. The coronal balance index was defined as the ratio of the side (left-right) with more weight to the opposite side, and the sagittal balance index was defined as the ratio of the side (front-back) with more weight to the opposite side. Ideally, 50% of the pressure is distributed in the left and right, front and back, which means that the values of coronal and sagittal balance index are close to 1.

##### SRS-22 questionnaire

The Scoliosis Research Society-22 (SRS-22) questionnaire was widely used to assess the quality of life in patients who had idiopathic scoliosis. The SRS-22 questionnaire contained 22 questions covering five domains: pain, self-image, function, mental health (five questions each), and management satisfaction (two questions), which has shown good validity and test-retest reliability [36]. Each item was scored from 1 (worst) to 5 (best), and the average of the five domains was used to calculate a total score.

### Statistical analysis

Data analysis was carried out using the Statistical Package for the Social Sciences (SPSS, Chicago, IL, USA) version 25.0, and the significance level was set at *p* < 0.05. The Shapiro-Wilk test was used to evaluate the normality of distribution, and Levene’s test was used to test the equality of variance. Descriptive data were presented as the mean ± SD for normally distributed data. The independent sample t-test between means and chi-square test for frequencies were used to assess differences between groups at baseline characteristics of participants. The treatment effectiveness with regard to this study’s three continuous variables (i.e., Cobb angle, ATR, SRS-22) was assessed using 2 × 3 (treatment group×time) repeated measures analysis of variance (ANOVA) tests.

## Results

**Fig. 4:**
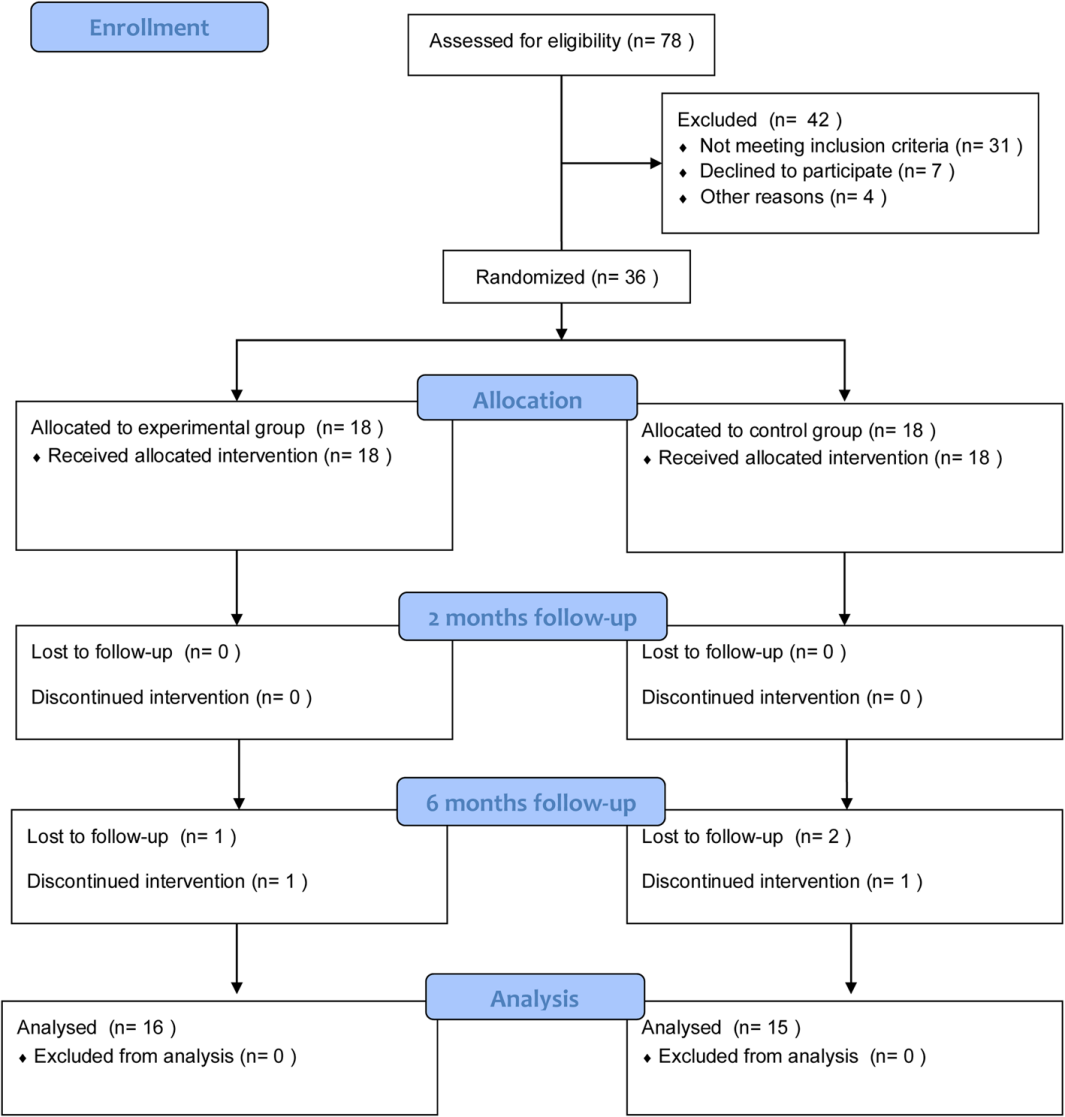
The flow of participants through the trial

The flow of participants through the trial is depicted in Fig. 4. Of the 36 randomly assigned participants, 16 cases in the experimental group and 15 cases in the control group were ultimately conducted for the analysis. Baseline clinical and demographic characteristics of the patients are shown in Table 1, and there was no difference between the groups in terms of general baseline characteristics (*p* > 0.05).

**Table 1:** Baseline characteristics of patients

**Characteristics**	**Experimental group (** ***n*** ** = 16)**	**Control group (** ***n*** ** = 15)**	***P*** ** value**
Age (years)	12.94 ± 1.39	13.67 ± 1.35	0.149
Sex (n)			
Female	13 (81.25%)	12 (80.0%)	0.999
Male	3 (18.75%)	3 (20.0%)	
Height (cm)	165.44 ± 5.45	165.87 ± 5.08	0.823
Weight (kg)	49.81 ± 7.02	53.73 ± 4.83	0.082
BMI (kg/m^2^)	18.16 ± 2.05	19.56 ± 1.91	0.059
Risser grade (n)			
1	2	0	0.158
2	5	8	
3	9	7	
King (n)			
1	3	4	0.826
2	7	5	
3	4	5	
4	2	1	
Cobb angle (°)	34.06 ± 8.43	34.40 ± 6.58	0.902

### Cobb angle

After two and six months of treatment, the Cobb angle in both groups significantly decreased compared with the baseline (*p* < 0.05). No significant group difference was found regarding the Cobb angle from baseline to the 2- and 6-month follow-up. No group-by-time interaction effects were observed. The results are shown in Table 2.

**Table 2:** Outcome measures by the group over time at the pre- and post-treatment assessments

**Variables**	**Groups**	**Baseline**	**2 months**	**6 months**	**Baseline** **VS** **2 months**	**Baseline** **VS** **6** **months**	**2 months** **VS** **6 months**	**Interaction** **(group and time)** **effect** **η2 (** ***P*** ** value)**	**Group effect** **η2 (** ***P*** ** value)**	**Time effect** **η2 (** ***P*** ** value)**
Cobb angle (°)	Insole	34.06 ± 8.43	24.13 ± 6.90	17.25 ± 5.89	< 0.001	< 0.001	< 0.001	0.029 (0.420)	0.013 (0.543)	0.893 (< 0.001)
	CG	34.40 ± 6.58	26.33 ± 7.66	19 ± 5.43	< 0.001	< 0.001	< 0.001			
ATR (°)	Insole	10.81 ± 3.19	9.25 ± 2.62	6.81 ± 1.56	< 0.001	< 0.001	< 0.001	0.080 (0.312)	0.026 (0.390)	0.849 (< 0.001)
	CG	11.13 ± 2.10	10.07 ± 1.79	7.67 ± 1.63	< 0.001	< 0.001	< 0.001			
Coronal balance index	Insole	1.27 ± 0.15	1.28 ± 0.14	1.15 ± 0.06	0.866	0.010	0.007	0.050 (0.229)	0.038 (0.292)	0.093 (0.059)
	CG	1.29 ± 0.22	1.27 ± 0.13	1.26 ± 0.13	0.663	0.671	0.798			
Sagittal balance index	Insole	1.36 ± 0.29	1.30 ± 0.18	1.22 ± 0.09	0.123	0.080	0.118	0.099 (0.233)	0.140 (0.038)	0.087 (0.278)
	CG	1.43 ± 0.26	1.38 ± 0.13	1.41 ± 0.10	0.433	0.820	0.436			
SRS-22										
Pain	Insole	4.14 ± 0.46	4.16 ± 0.42	4.23 ± 0.35	0.787	0.276	0.386	0.007 (0.813)	0.007 (0.646)	0.063 (0.150)
	CG	4.21 ± 0.49	4.19 ± 0.47	4.32 ± 0.38	0.737	0.282	0.065			
Self-image	Insole	3.20 ± 0.46	3.31 ± 0.39	3.35 ± 0.37	0.479	0.308	0.682	0.037 (0.586)	< 0.001 (0.951)	0.091 (0.264)
	CG	3.29 ± 0.53	3.21 ± 0.40	3.37 ± 0.39	0.686	0.677	0.104			
Function	Insole	4.54 ± 0.38	4.71 ± 0.26	4.76 ± 0.27	0.164	0.076	0.468	0.065 (0.389)	0.021 (0.441)	0.180 (0.062)
	CG	4.61 ± 0.30	4.56 ± 0.30	4.67 ± 0.25	0.623	0.524	0.056			
Mental health	Insole	3.61 ± 0.38	3.51 ± 0.35	3.51 ± 0.38	0.348	0.355	0.999	0.018 (0.596)	0.106 (0.073)	0.092 (0.060)
	CG	3.50 ± 0.26	3.23 ± 0.38	3.37 ± 0.24	0.067	0.164	0.205			
Satisfaction	Insole	---	4.25 ± 0.37	4.5 ± 0.41	---	---	0.072	0.042 (0.269)	< 0.001 (0.934)	0.116 (0.061)
	CG	---	4.33 ± 0.36	4.40 ± 0.28	---	---	0.499			

*Insole* Experimental group, *CG *Control group

### Angle of trunk rotation (ATR)

The ATR in both groups significantly decreased at two months and six months (*p* < 0.05) (Table 2). Intergroup comparison revealed no significant difference between the groups in the angle of trunk rotation at the two- and six-month follow-up. There were no significant group-by-time interactions for the ATR.

### Coronal balance index and sagittal balance index

At baseline, the values of coronal balance and sagittal balance index deviated from 1 for both groups, implying that AIS patients might have postural instability and center of gravity deviation. After the intervention, the value of sagittal balance index significantly decreased at six months compared to the control group (*p* < 0.05). This difference was not observed at two months. The significant difference in coronal balance index was observed at six months compared to baseline in the experimental group (*p* < 0.05), and no significant inter-group differences were found from baseline to the two- and six-month follow-up. The results are displayed in Table 2.

### SRS-22

No difference was observed in the groups in the SRS-22 questionnaire of its five domains (Table 2).

## Discussion

At present, there are few clinical trials investigating the impact of insoles on AIS. This study aimed to evaluate the effects of insoles on the spinal curvature of patients with moderate AIS. The study results demonstrated that combined insoles with bracing and exercises had similar effects on Cobb angle, angle of trunk rotation, and quality of life compared to traditional bracing and exercise interventions during two- and six months of treatment in patients with moderate AIS, but improved the coronal balance index and sagittal balance index at six months. This suggested that 3D personalized insoles in our study cannot play a synergistic role with traditional interventions in improving spinal deformity in patients with moderate AIS, but might have the potential of improving the static balance in the mid-and-long term. In the meantime, our study reconfirmed the effectiveness of bracing combined with exercises in treating AIS and strengthened the previous evidence.

In patients with AIS, the asymmetry of the spine due to scoliosis might result in an abnormal force on the pelvis. When the load of the upper limbs and spine was transferred to the lower limbs, the load on the lower limbs would be changed, which could lead to an asymmetric force on the left and right sides of the trunk, pelvis, lower limbs, and feet [37–39]. The asymmetric load and force on both sides of the body would lead to skeletal growth disorders, aggravating scoliosis. This could create a vicious circle that could result in the progression of AIS, especially when AIS occurs during a growth spurt. In addition, AIS patients were also associated with biomechanical abnormalities of the lower extremities, such as flat feet, high-arched feet, and leg length discrepancy. Therefore, we cannot only consider a single aspect of the spine in formulating a protocol for the treatment of AIS, but also consider the abnormal biomechanics of the pelvis, lower limb, and foot, etc., breaking the original abnormal pattern of the musculoskeletal system in patients with scoliosis and preventing further deterioration [40]. Therefore, this study aimed to stop this vicious cycle by adjusting the asymmetrical force on both sides of the body through insoles so that the asymmetrical force could be balanced and the spine could grow normally. However, our findings were consistent with prior research that showed no statistically significant effect of proprioceptive insoles on spinal curvature in patients with slight idiopathic scoliosis [23].

The initial hypotheses were expected based on evidence from previous studies that insoles are known to positively impact foot disorders, improve neurological disabilities, induce spinal postural changes, and reduce chronic musculoskeletal imbalances [22]. Additionally, some researchers had identified that insoles had a positive impact on muscular tension, joint malposition, pelvic misalignment, and the spinal curvature in the sagittal plane could be positively influenced by specific stimulations and relaxations of the plantar foot muscles together with the increase of low muscle tonus and reduction of high muscle tonus [41]. Unfortunately, in recent years, the effect of insoles on sagittal balance has not been well described due to the lack of clinical studies. The results of this study showed that insoles had no positive effect on trunk deformity, but had a positive effect on improving the coronal balance index and sagittal balance index at six months, which might imply insoles have the potential of improving the static balance and promoting postural adjustment of AIS patients.

A previous study revealed that adolescents with a progressive form of idiopathic scoliosis had fair postural stability compared to healthy peers [6]. For insoles used in this study, we increased the stress points or cut off the load-free parts of the insole, including personalized increased arch support, adding wedge blocks with different heights to the heel for adjusting the heel in a neutral position, controlling the degree of forefoot inversion and eversion, and bilateral lower limb length compensation, etc. The above allowed the patient’s body to be adjusted in a neutral position and gave sufficient sensory input to facilitate the postural adjustment. Leg length discrepancy (LLD) is very common in patients with scoliosis, and insoles can be used to correct it. Some studies indicate that treatment needs to be implemented when LLD is above 1 cm; however, other studies are more conservative and believe that treatment is only required when LLD is above 2 cm [42, 43]. The correction of leg length discrepancy could reduce the curves of patients with scoliosis [37]. Patients included in our study had bilateral lower extremity inequalities of less than 1 cm. It is unclear whether insoles can effectively improve spinal deformities or balance in the short term while LLD is above 1 cm.

The negative effects of scoliosis on quality of life and related constructs such as psychosocial health have been reported in the literature [44]. The main concern of patients with scoliosis is anxiety, which developed due to a three-dimensional deformity. The correction of the cosmetic deformity is a primary goal of treatment, as reported in a consensus by SOSOR [45]. Negrini et al. revealed in a systematic review that bracing for AIS was able to prevent curve progression and could not improve quality of life and back pain caused by the spinal curvature [46]. Our results were consistent with these studies. The discomfort that patients may experience due to the use of braces and insoles can significantly reduce scores related to quality of life, which may be greatly improved in the future if the patient’s trunk deformity is minimized and extrinsic interventions are discontinued. It was worth mentioning that patients in both groups reported satisfaction with treatments in the subdomains of SRS-22.

There were some limitations of this study that needed to be taken into account. Firstly, treatment compliance was not considered in the analysis. Although we have instructed the guardians to take photos or videos at regular intervals and send them to us, the impact of compliance with daily home exercises on the outcome could not be thoroughly assessed. Secondly, we only performed a follow-up of 6 months, as the insole may have a distortion during long-term use due to weight-bearing, which may affect the treatment effect. Thirdly, the curvature pattern in the study was classified by the King classification system, and patients with types one to four in this study may have added some bias. Future studies could be specifically limited to a particular type. Fourthly, although 3D personalized insoles cannot reduce the Cobb angle and angle of trunk rotation of patients with moderate AIS in our study, it was still interesting to use finite element analysis to explore the insole optimization. Finite element modeling can become an efficient tool for an in-depth understanding of the insole design on stress distribution, displacement and bone rotation of the spine. Unfortunately, finite element analysis was missing in this study.

## Conclusion

In conclusion, the results indicated that bracing combined with exercise could reduce curve progression and the angle of trunk rotation in patients with moderate AIS, which strengthened the previous evidence. 3D personalized insoles cannot reduce patients with moderate AIS’ Cobb angle and angle of trunk rotation but might have the potential to improve postural stability.

## Data Availability

The datasets generated and/or analyzed during the current study are not publicly available due to the privacy and ethical concerns of minors but are available from the corresponding author on reasonable request via the e-mail zm1455@163.com.

## References

[1] Altaf F, Gibson A, Dannawi Z, Noordeen H (2013). Adolescent idiopathic scoliosis. BMJ.

[2] Meng ZD, Li TP, Xie XH, Luo C, Lian XY, Wang ZY (2017). Quality of life in adolescent patients with idiopathic scoliosis after brace treatment: A meta-analysis. Med (Baltim).

[3] Addai D, Zarkos J, Bowey AJ (2020). Current concepts in the diagnosis and management of adolescent idiopathic scoliosis. Childs Nerv Syst.

[4] Weinstein SL, Dolan LA, Cheng JC, Danielsson A, Morcuende JA (2008). Adolescent idiopathic scoliosis. Lancet.

[5] Daryabor A, Arazpour M, Sharifi G, Bani MA, Aboutorabi A, Golchin N (2017). Gait and energy consumption in adolescent idiopathic scoliosis: A literature review. Ann Phys Rehabil Med.

[6] Wiernicka M, Kotwicki T, Kamińska E, Łochyński D, Kozinoga M, Lewandowski J (2019). Postural Stability in Adolescent Girls with Progressive Idiopathic Scoliosis. Biomed Res Int.

[7] Karimi MT, Kavyani M, Kamali M (2016). Balance and gait performance of scoliotic subjects: A review of the literature. J Back Musculoskelet Rehabil.

[8] Kuznia AL, Hernandez AK, Lee LU (2020). Adolescent Idiopathic Scoliosis: Common Questions and Answers. Am Fam Physician.

[9] Ceballos Laita L, Tejedor Cubillo C, Mingo Gómez T, Jiménez Del Barrio S (2018). Effects of corrective, therapeutic exercise techniques on adolescent idiopathic scoliosis. A systematic review. Arch Argent Pediatr.

[10] Fan Y, Ren Q, To MKT, Cheung JPY (2020). Effectiveness of scoliosis-specific exercises for alleviating adolescent idiopathic scoliosis: a systematic review. BMC Musculoskelet Disord.

[11] Gao C, Zheng Y, Fan C, Yang Y, He C, Wong M (2019). Could the Clinical Effectiveness Be Improved Under the Integration of Orthotic Intervention and Scoliosis-Specific Exercise in Managing Adolescent Idiopathic Scoliosis?: A Randomized Controlled Trial Study. Am J Phys Med Rehabil.

[12] Negrini S, Minozzi S, Bettany-Saltikov J, Chockalingam N, Grivas TB, Kotwicki T (2015). Braces for idiopathic scoliosis in adolescents. Cochrane Database Syst Rev.

[13] Smania N, Picelli A, Romano M, Negrini S (2008). Neurophysiological basis of rehabilitation of adolescent idiopathic scoliosis. Disabil Rehabil.

[14] Yagci G, Yakut Y (2019). Core stabilization exercises versus scoliosis-specific exercises in moderate idiopathic scoliosis treatment. Prosthet Orthot Int.

[15] Tian F, Yang YF, Ding T (2018). Correlation between adolescent scoliosis and biomechanical factors. Chin J Rehabil Theory Pract.

[16] Zhu F, Hong Q, Guo X, Wang D, Chen J, Zhu Q (2021). A comparison of foot posture and walking performance in patients with mild, moderate, and severe adolescent idiopathic scoliosis. PLoS ONE.

[17] Gong L, Fang L, Ye XL, Fan YS, Jiang YQ, Tong PJ (2020). Progress on the treatment of adolescent idiopathic scoliosis. China J Orthop Trauma.

[18] Guy A, Coulombe M, Labelle H, Rigo M, Wong MS, Beygi BH (2022). Biomechanical Effects of Thoracolumbosacral Orthosis Design Features on 3D Correction in Adolescent Idiopathic Scoliosis: A Comprehensive Multicenter Study. Spine (Phila Pa 1976).

[19] Xu R, Wang Z, Ren Z, Ma T, Jia Z, Fang S (2019). Comparative Study of the Effects of Customized 3D printed insole and Prefabricated Insole on Plantar Pressure and Comfort in Patients with Symptomatic Flatfoot. Med Sci Monit.

[20] Zhang XY, Xing XY, Huo HF (2020). Design principle and biomechanical function of orthopedic insoles. J Clin Rehabil Tis Eng Res.

[21] Choo YJ, Park CH, Chang MC (2020). Rearfoot disorders and conservative treatment: a narrative review. Ann Palliat Med.

[22] Dankerl P, Keller AK, Häberle L, Stumptner T, Pfaff G, Uder M (2016). Effects on posture by different neuromuscular afferent stimulations and proprioceptive insoles: Rasterstereographic evaluation. Prosthet Orthot Int.

[23] Noll C, Steitz V, Daentzer D (2017). Influence of proprioceptive insoles on spinal curvature in patients with slight idiopathic scoliosis. Technol Health Care.

[24] Lewinson RT, Stefanyshyn DJ (2016). Wedged Insoles and Gait in Patients with Knee Osteoarthritis: A Biomechanical Review. Ann Biomed Eng.

[25] Steinberg N, Waddington G, Adams R, Karin J, Begg R, Tirosh O (2016). Can textured insoles improve ankle proprioception and performance in dancers?. J Sports Sci.

[26] Xiang L, Mei Q, Wang A, Shim V, Fernandez J, Gu Y (2022). Evaluating function in the hallux valgus foot following a 12-week minimalist footwear intervention: A pilot computational analysis. J Biomech.

[27] Schulz KF, Altman DG, Moher D, Group CONSORT (2011). CONSORT 2010 statement: updated guidelines for reporting parallel group randomised trials. Int J Surg.

[28] Seifert J, Thielemann F, Bernstein P (2016). Adoleszente idiopathische Skoliose: Leitfaden für die praktische Anwendung [Adolescent idiopathic scoliosis : Guideline for practical application]. Orthopade.

[29] Weinstein SL, Dolan LA, Wright JG, Dobbs MB (2013). Effects of bracing in adolescents with idiopathic scoliosis. N Engl J Med.

[30] Gür G, Ayhan C, Yakut Y (2017). The effectiveness of core stabilization exercise in adolescent idiopathic scoliosis: A randomized controlled trial. Prosthet Orthot Int.

[31] Zaina F, Fusco C, Atanasio S, Negrini S (2011). The SPoRT concept of bracing for idiopathic scoliosis. Physiother Theory Pract.

[32] Yagci G, Ayhan C, Yakut Y (2018). Effectiveness of basic body awareness therapy in adolescents with idiopathic scoliosis: A randomized controlled study1. J Back Musculoskelet Rehabil.

[33] Romano M, Minozzi S, Bettany-Saltikov J, Zaina F, Chockalingam N, Kotwicki T (2012). Exercises for adolescent idiopathic scoliosis. Cochrane Database Syst Rev.

[34] Jacquot F, Charpentier A, Khelifi S, Gastambide D, Rigal R, Sautet A (2012). Measuring the Cobb angle with the iPhone in kyphoses: a reliability study. Int Orthop.

[35] Côté P, Kreitz BG, Cassidy JD, Dzus AK, Martel J (1998). A study of the diagnostic accuracy and reliability of the Scoliometer and Adam’s forward bend test. Spine (Phila Pa 1976).

[36] Cheung KM, Senkoylu A, Alanay A, Genc Y, Lau S, Luk KD (2007). Reliability and concurrent validity of the adapted Chinese version of Scoliosis Research Society-22 (SRS-22) questionnaire. Spine (Phila Pa 1976).

[37] Rothschild D, Ng SY, Ng YLE (2020). Indications of sole lift and foot orthoses in the management of mild idiopathic scoliosis-a review. J Phys Ther Sci.

[38] Daryabor A, Arazpour M, Samadian M, Veiskarami M, Ahmadi Bani M (2017). Efficacy of corrective spinal orthoses on gait and energy consumption in scoliosis subjects: a literature review. Disabil Rehabil Assist Technol.

[39] Kim K, Mullineaux DR, Jeon K (2019). A Comparative Study of Spinal Deformity and Plantar Pressure according to the Static Standing Posture of Female Adolescents with or without Idiopathic Scoliosis. Iran J Public Health.

[40] Zhao Y, Wang CH, Li D, Han XL, Wang YJ, Li TT (2018). The study of orthopedic insole for posture adjustment of adolescent idiopathic scoliosis. Chin J Phys Med Rehabil.

[41] Müller-Gliemann C, Drerup B, Osada N, Wetz HH (2006). The influence of proprioceptive insoles (Bourdiol) on the sagittal curvature and inclination of the trunk. Orthopade.

[42] Vogt B, Gosheger G, Wirth T, Horn J, Rödl R (2020). Leg Length Discrepancy- Treatment Indications and Strategies. Dtsch Arztebl Int.

[43] Shi Y, Pang H, Xu H, Li X, Cao Y, Merryweather A (2022). Effects of orthotic insole on gait patterns in children with mild leg length discrepancy. Gait Posture.

[44] Lonner B, Yoo A, Terran JS, Sponseller P, Samdani A, Betz R (2013). Effect of spinal deformity on adolescent quality of life: comparison of operative scheuermann kyphosis, adolescent idiopathic scoliosis, and normal controls. Spine (Phila Pa 1976).

[45] Negrini S, Donzelli S, Aulisa AG, Czaprowski D, Schreiber S, de Mauroy JC (2018). 2016 SOSORT guidelines: orthopaedic and rehabilitation treatment of idiopathic scoliosis during growth. Scoliosis Spinal Disord.

[46] Negrini S, Minozzi S, Bettany-Saltikov J, Chockalingam N, Grivas TB, Kotwicki T (2016). Braces for Idiopathic Scoliosis in Adolescents. Spine (Phila Pa 1976).

